# RACIAL AND ETHNIC DISPARITIES IN SOCIAL ISOLATION AND 11-YEAR DEMENTIA RISK AMONG OLDER ADULTS IN THE UNITED STATES

**DOI:** 10.1093/geroni/igad104.3546

**Published:** 2023-12-21

**Authors:** Jason Grullon, Daniel Soong, Roger Wong

**Affiliations:** SUNY Upstate Medical University, Syracuse, New York, United States; SUNY Upstate Medical University, Syracuse, New York, United States; SUNY Upstate Medical University, Syracuse, New York, United States

## Abstract

Social isolation has been linked to cognitive impairment. Research on this relationship, however, remains limited because validated social isolation measures have been underutilized when examining dementia risk, while also considering differences among racial-ethnic groups. To address this gap, we analyzed 11 years of data (2011–2021) from the National Health and Aging Trends Study to examine the interplay between social isolation, race-ethnicity, and dementia. We constructed a longitudinal score using a validated social isolation variable for our sample of 6,155 community-dwelling U.S. adults aged 65 years and older. After applying sampling weights and imputing missing data, our analysis revealed a higher average longitudinal frequency of social isolation among Black (27.6%), Hispanic (26.6%), and Asian (21.0%) older adults compared to non-Hispanic White (19.1%) respondents during the 11-year period (t=-7.35, p<.001). Cox regression models indicated higher frequency of social isolation was significantly associated with a 45% increased dementia risk after adjusting for sociodemographic covariates (adjusted Hazard Ratio [aHR]=1.45, 95% CI=1.14-1.84, p<.01), but this association was non-significant after further adjusting for health covariates (aHR=1.16, 95% CI=0.92-1.46, p=.20). Race-ethnicity was also not a significant moderator in the association between social isolation and dementia. Our results suggest older adults of color experience higher longitudinal frequency of social isolation. Despite the elevated dementia risk we observed among older adults with higher social isolation, race-ethnicity did not moderate this relationship. Future research is needed to investigate the underlying mechanisms contributing to racial-ethnic disparities for social isolation, and develop targeted interventions to mitigate the associated risk for dementia.

